# Supplementary material for: MetaQTL: a package of new computational methods for the meta-analysis of QTL mapping experiments
Source: BMC Bioinformatics. 2007 Feb 8;8:49. doi: 10.1186/1471-2105-8-49 (PMC1808479; doi:10.1186/1471-2105-8-49)
Supplement: Additional File 7 — MetaQTL Package : jar file and tutorial. This Zip archive contains both the MetaQTL JAR file and the files of the tutorial. [file 1471-2105-8-49-S7.zip › org.inra.metaqtl/doc/org/metaqtl/algo/class-use/MetaAlgorithm.html]

Uses of Class org.metaqtl.algo.MetaAlgorithm


|  |  |  |
| --- | --- | --- |
| **Overview** \| **Package** \| **Class** \| **Use** \| **Tree** \| **Deprecated** \| **Index** \| **Help** | |  |
| PREV   NEXT | **FRAMES**    **NO FRAMES**     **All Classes** |  |


---


## **Uses of Class org.metaqtl.algo.MetaAlgorithm**

| Packages that use MetaAlgorithm | |
| --- | --- |
| **org.metaqtl.algo** |  |

Uses of MetaAlgorithm in org.metaqtl.algo

| Subclasses of MetaAlgorithm in org.metaqtl.algo | |
| --- | --- |
| `class` | `ClustAlgorithm` |
| `class` | `MetaMapAlgorithm`             This class is dedicated to the construction of a consensus genetic map from a set of genetic maps based on a Weigthed Least Square approach. |
| `class` | `QtlClustAlgorithm`             This class defines methods to perform a QTL meta-analysis on a genetic map by applying a clustering of QTL based on a gaussian mixture model and the EM-algorihtm. |
| `class` | `QtlProjAlgorithm`             This class defines some methods to project QTL positions from a genetic map to another. |
| `class` | `QtlTreeAlgorithm`             This class defines methods to perform agglomerative hierarchical QTL clustering. |

---


|  |  |  |
| --- | --- | --- |
| **Overview** \| **Package** \| **Class** \| **Use** \| **Tree** \| **Deprecated** \| **Index** \| **Help** | |  |
| PREV   NEXT | **FRAMES**    **NO FRAMES**     **All Classes** |  |


---
